# The signing body: extensive sign language practice shapes the size of hands and face

**DOI:** 10.1007/s00221-021-06121-9

**Published:** 2021-05-24

**Authors:** Laura Mora, Anna Sedda, Teresa Esteban, Gianna Cocchini

**Affiliations:** 1 Department of Psychology, Goldsmiths, University of London, London, UK; 2 Department of Psychology, Heriot-Watt University, Edinburgh, UK

**Keywords:** Body representation, Body metrics, Sign language, Hand, Face

## Abstract

The representation of the metrics of the hands is distorted, but is susceptible to malleability due to expert dexterity (magicians) and long-term tool use (baseball players). However, it remains unclear whether modulation leads to a stable representation of the hand that is adopted in every circumstance, or whether the modulation is closely linked to the spatial context where the expertise occurs. To this aim, a group of 10 experienced Sign Language (SL) interpreters were recruited to study the selective influence of expertise and space localisation in the metric representation of hands. Experiment 1 explored differences in hands’ size representation between the SL interpreters and 10 age-matched controls in near-reaching (Condition 1) and far-reaching space (Condition 2), using the localisation task. SL interpreters presented reduced hand size in near-reaching condition, with characteristic underestimation of finger lengths, and reduced overestimation of hands and wrists widths in comparison with controls. This difference was lost in far-reaching space, confirming the effect of expertise on hand representations is closely linked to the spatial context where an action is performed. As SL interpreters are also experts in the use of their face with communication purposes, the effects of expertise in the metrics of the face were also studied (Experiment 2). SL interpreters were more accurate than controls, with overall reduction of width overestimation. Overall, expertise modifies the representation of relevant body parts in a specific and context-dependent manner. Hence, different representations of the same body part can coexist simultaneously.

## Introduction

Distortions in body representation, such as perceiving parts of our body shorter or wider, are part of healthy experience. Using the well-known localisation task, researchers have been able to collect information on the body model of the hands (e.g., Longo and Haggard 2012), the face (Longo and Holmes 2020; Mora et al. 2018), and lower limbs (Stone et al. 2018), which are intrinsically distorted. More specifically, fingers are underestimated in length, whilst hands are overestimated in width (e.g., Longo and Haggard 2010). Similarly, the face is perceived much wider than its real size, whilst length is slightly underestimated (Mora et al. 2018). Hand distortions are assumed to be quite robust and resistant to changes on the type of instructions (Longo 2018), task modality (Ambroziak et al. 2018; Peviani and Bottini 2018), or hand orientation (e.g., Longo and Haggard 2010; Saulton et al. 2016). However, some studies have shown how the extent of the distortion can be modulated by multisensory information, such as positional changes (Longo 2015); vision (Longo 2014); tool use and type of action (Romano et al. 2019); sound (Tajadura-Jiménez et al. 2017), and attentional impairments (Caggiano et al. 2020), confirming that the representation of the body is highly malleable (Ambron et al. 2018; Medina and Coslett 2016; Medina et al. 2010).

A growing body of evidence demonstrates that long-term training and expertise can modulate our body representation. For example, expert magicians have an improved perception of finger lengths (Cocchini et al. 2018), whilst professional dancers show better capacity for proprioceptive localisation of their hand (Jola et al. 2011) and single joints (Kuni and Schmitt 2004; Ramsay and Riddoch 2001). Interestingly, the effect of practice not only translates into behavioural differences in perceptual performance, but also in cortical excitability (Hallett 2001). That is, structural (Meier et al. 2016) and connectivity brain changes are found in expert dancers (Burzynska et al. 2017), whereas improved dexterity of fingers through training brings cortical long-term activation adjustments in the motor cortex (Kami et al. 1995). On the contrary, reduced use is associated with a shrinkage of representation due to decreased cortical excitability, such as in the case of cast use (Liepert et al. 1995; Lissek et al. 2009), or short-term immobilization (Opie et al. 2016). These structural and functional changes are seen even after short-lasting tactile training for Braille reading in healthy volunteers (Debowska et al. 2016).

Interestingly, anatomical cortical changes also appear due to long-term expertise when the body is used for communication purposes, such as in Sign Language (SL). Indeed, fine motor control of the hands for signing causes structural differences in the volume of the hand knob (Allen et al. 2013; Penhune et al. 2003), an area that includes the sensorimotor representation of the hand (Sastre-Janer 1998). Expert SL users show differences in cortical thickness (Hervais-Adelman et al. 2017), hyperconnectivity in prefrontal regions during resting state (Klein et al. 2018), and more bilateral activation when processing emotional facial expressions (Emmorey and McCullough 2009). Improved somatosensory processing is supported by activation of the left superior parietal lobule, to monitor the SL output through proprioception, and supramarginal gyrus, for the selection of hand configurations and locations (Emmorey et al. 2007) and motor planning of hand movements (Hesse et al. 2006). The latter, instead, is not active for oral word production (Indefrey and Levelt 2004). Furthermore, parietal lobes are engaged in spatial processing, but also in the monitoring of body localisation and positions (Campbell et al. 2007), and storage of the structural and sensorimotor body representation (Corradi-Dell’Acqua et al. 2009; Hashimoto and Iriki 2013; Tamè et al. 2017). Configuration and location processing of hands in SL are associated with left hemisphere superior and inferior parietal lobe activation (MacSweeney et al. 2002), whilst other studies have found bilateral recruitment of parietal cortices (Emmorey 2006), areas presumed to store the structural representation of the body (Corradi-Dell’Acqua et al. 2009; Tamè et al. 2017) and own body size perception (Hashimoto and Iriki 2013). Hence, SL prolonged practice may influence the way the size of these body parts is represented.

Similarly, the linguistic experience in SL translates into better perceptual abilities. When interpreting, SL practitioners need to simultaneously process heard language, maintaining the message in short-term memory at the same time as signing the message coherently with the language format used (Klein et al. 2018). This experience is associated with functional gains in visuo-spatial abilities for mental rotation (Emmorey et al. 1993; Keehner and Gathercole 2007), and in generating mental images (Emmorey et al. 1993). Moreover, SL use improves working memory, as addressees need to retain visual sequences of hand shapes, and face and body movements to convey meaning (Arnold and Mills 2001). Furthermore, signers rely on somatosensory processing for signing processes (Emmorey et al. 2009a, b), associated with better overall kinesthetics and visuo-motor skills, as seen in magicians (Cavina-Pratesi et al. 2011). Similarly, long use of SL results in “enhanced processing of hands” in the left hemisphere (dominant for language), even when not signing (Mitchell 2017, p. 159).

Proficient SL users need to be able to move their hands rapidly and precisely and use their face simultaneously to convey meaning (Bettger et al. 1997; Muir and Richardson 2005). Moreover, SL users have to coordinate a range of positions, movements, and locations altogether. Particularly, handshapes (configurations of fingers and palm) have to be combined with changes on location (position of the hand respective to the other hand, face, trunk, or in signing space), movement (action performed), and orientation (direction of the palm) to provide meaning (Sehyr and Cormier 2016). For example, in British SL (BSL), vowels are spelled by pointing to the fingertips of the non-dominant hand (Sutton-Spence et al. 1990). The words ‘pig’ and ‘witch’ are both signed at the nose but with different handshapes, whilst the words ‘name’ and ‘afternoon’ have a shared handshape, only varying on their location (head and chin, respectively) (MacSweeney et al. 2008). Furthermore, the face is not only used as location for manual gestures; SL users need to identify and distinguish quick facial expressions that have linguistic or emotional connotations for the perception of meaning (Bettger et al. 1997; Emmorey and McCullough 2009). Indeed, negation in BSL is indicated with non-manual gestures (headshake, furrowed brow, or frowning) (Campbell et al. 2007), whilst mouth configurations indicate adverbial meaning when accompanied by American SL (ASL) verbs (Emmorey and McCullough 2009).

Moreover, these manual and facial actions are space-dependent, as these are performed within a circumscribed three-dimensional area in near-reaching space that mostly extends from the forehead to the waist, to the front of the face and chest, and laterally beyond the elbows (Arnold and Mills 2001; Emmorey 2001). Indeed, there is a close link between space and body representation. For example, studies have shown how the perceived length of the arms or their affordances can extend the size of peripersonal space (Longo and Lourenco 2007) or reduce it (Lourenco and Longo 2009), as the body height does (D’Angelo et al. 2019). Hence, size perception of ones’ own body appears to be linked to space and to the possibilities of action (Bassolino et al. 2015; D’Angelo et al. 2018). As seen before, spatial organisation is characteristic of visual-gestural languages (Bellugi and Klima 2015), and this may have an effect on the metric representation of SL practitioners’ body. Moreover, visuo-motor-proprioceptive cross-modal interactions are intrinsic to hand use (Korb et al. 2017). The evidence above makes a strong case to study the representation of hands in different portions of space (i.e., near- or far-reaching space). If the metric representation of hands is associated with the manual workspace and type of expertise, the impact of expertise will be strongly related to the spatial domain. Therefore, SL interpreters will show different metric representation of hands only in near-reaching space, whereas no differences should be found in far-reaching. Hence, in Experiment 1, the representation of the hands in ‘near’ and ‘far’ reaching space was explored, to elucidate any differences on representation due to expertise and space localisation.

Additionally, SL uses the face for non-manual gestures and expressions, and this use may also influence face representation, in such a way that distortions are reduced. Hence, a second aspect of this study is to further understand if expertise, such as signing, can have similar effect on different body parts within the same expert group. Experiment 2 aims to address whether signing has a relevant impact on the metric representation not only of the hands, but also of the face (Experiment 2).

## Experiment 1

### Methods and procedures

#### Participants

An a priori power analysis was run to determine the required sample size by using G* Power 3.1 (Faul et al. 2009). The effect sizes (Cohen’s *d*) from a previous study with baseball experts were considered for this calculation (Coelho et al. 2019). In this case, the lowest effect size for the independent *t* tests between groups was 1.65. A power analysis for the difference between two independent means (two groups) with an effect size of 1.65, alpha of 0.05, and power of 0.8 indicated the adequate sample size would be of 7 per group (14 overall).

Twenty participants (16 females and 4 males) between 24 and 63 years of age (mean age = 40.85 years, SD = 10.8) took part in this study. Ten of them (8 females and 2 males) were recruited as expert SL interpreters (mean age = 45.4, SD = 8.69) from SL associations and educational settings, such as Heriot-Watt University, and through snowball sampling. SL use is associated with different activation patterns in the brain; however, plasticity of these networks varies depending on the hearing status of the user, when language acquisition occurs and the levels of exposure to the SL (Campbell et al. 2007). For example, studies have shown thicker white matter connections between auditive regions in hearing users, when compared with Deaf users (Emmorey et al. 2003). To control for variability, only bimodal signers were recruited (i.e., hearing bilingual signers with both oral and signed languages). These participants were required to have at least 3 years of professional practice, with over 10 h of use per week, and at least 3 years of previous formal training.

Handedness was assessed with the Edinburgh Handedness Inventory (Oldfield 1971), to consider if there were effects in hand size representation associated with dominance. Values range from − 1 to 1, with scores below − 0.5 indicating left-handedness; scores between − 0.5 to + 0.5 indicating ambidexterity; and scores over + 0.5 indicating right-handedness. From the ten participants, two were ambidextrous (scores = − 0.32 and − 0.09, respectively), whilst eight were right-handed. All SL professionals used the right hand as the dominant hand for signing. Demographic details, handedness, and expertise details are reported in Table 1.

**Table 1:** Participants’ demographics

	SL group (*N* = 10)	SL group (*N* = 10)
Age (years)		
Mean	45.4	36.3
SD	8.69	11.16
Range	35–60	24–63
Post-secondary school education (years)		
Mean	5.6	5.2
SD	2.32	2.15
Range	2–9	2–8
Edinburgh Handedness Inventory		
Mean	0.67	0.68
SD	0.48	0.6
Range	− 0.09 to 1	− 1 to 1
*Degree of expertise as sign language practitioner*		
Years of practice		
Mean	20.34	–
SD	9.89	
Range	3.5–39	
Practice per week (h)		
Mean	38.4	–
SD	25.69	
Range	15–84	

Demographic and handedness characteristics with the SL interpreters’ degree of expertise (SD = standard deviation)

The other ten participants acted as control group, and they were matched by age (M = 36.3, SD = 11.16), gender (8 females), and handedness (one left handed participant; score = − 1) (see Table 1). Analyses did not show any differences by age [*t*(18) = − 2.04, *p* = 0.06, *d* = 0.91], handedness [*t*(18) = 0.06, *p* = 0.96, *d* = 0.03], or education [*t*(18) = − 0.4, *p* = 0.69, *d* = 0.18] between groups. Frequency of females in each group was identical (see Table 1). None of the control participants had practiced or learned SL or used their hands or faces for any other professional or artistic purposes requiring specific training and ability. Goldsmiths Research Committee approved this study, and it was carried out in accordance with the Helsinki Declaration. All participants provided written informed consent.

#### Hand localisation task and procedure

**Fig. 1:**
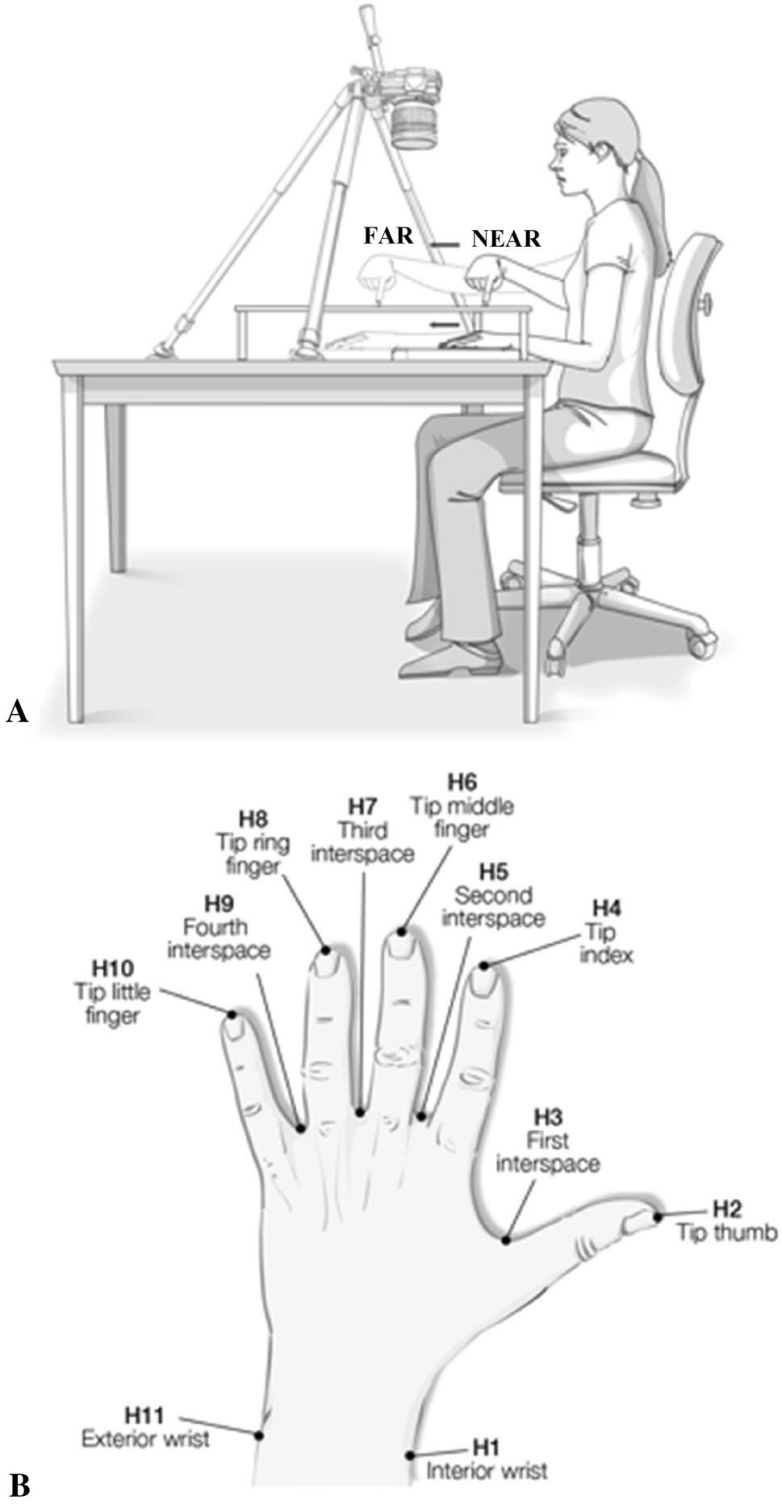
Hand localisation task. Picture of hand setting showing hand position in near and in far conditions (**a**); and illustration of hand’s and wrist’s landmarks (**b**)

A modified version of the hand localisation task (Longo and Haggard 2012) was used in this study. A horizontal transparent Perspex board (50 × 55 cm) resting on four wooden posts (each 10 cm high) was positioned on a table in front of the participant. A remote-controlled camera (Nikon D3200) was used to record participants’ responses, positioned over the board (90 cm high) with a tripod, with its focus aligned to the centre of the board. A small 20 × 20 cm white canvas was positioned underneath, onto which the participants rested their hands (one at a time). This canvas was positioned at two different distances from the body for two different conditions: ‘near’ and ‘far’ distances. In the ‘near’ condition, the canvas was placed at a distance of around 15 cm to the body, in such a way that the canvas was just at the edge of the table, allowing participants to only position the hand and wrist under the Perspex board. The ‘near’ condition would therefore take place within the three-dimensional signing space. In contrast, in the ‘far’ condition, the canvas was moved further forward, at the edge of the individual’s reaching space (at about 45 cm from the body), and it would be a space that both SL interpreters and controls rarely use in their day-to-day. Participants rested their elbow on the table, whilst extending their arm as far as it was comfortable underneath the board (see Fig. 1a). Both conditions were counterbalanced. A measuring tape was attached to the top and right edges of the Perspex board, to allow later conversion of pixels into centimetres for each pointing response. Participants were sat in front of a table whilst keeping their eyes closed. One hand (either the right or left, counterbalanced) was positioned underneath the Perspex board, and on top of the white canvas frame (see Fig. 1a). The middle finger was aligned with the participant’s body midline, whilst the other fingers were spread out comfortably. Participants were asked to keep the hand under the board completely still, whilst using the other hand’s index finger to point to the required locations. A small dot (around 1 mm diameter) was drawn on the tip of the index’s fingernails as reference for later analyses.

A total of 11 hand landmarks were read aloud (see Fig. 1b), one at a time (5 fingertips, 4 interspaces and the two sides of the wrist’s bones, ulna and radius). Participants were previously trained to understand the different labels for each landmark by identifying these on a schematic drawing. Landmarks were given in order, starting either from the interior bone of the wrist, the radius (H1 landmark, see Fig. 1b); or from the exterior bone, the ulna (landmark H11). The starting landmark was randomized across participants. Each landmark was requested twice with a total of 22 trials per hand (final total of 88 pictures considering two hands and two conditions). Participants were required to point to each landmark on top of the board by using their index fingers. They were allowed to make pointing adjustments to avoid ballistic responses’ variability (Mora et al. 2018; Kammers et al. 2009; Króliczak et al. 2006). Once the landmark was located, a picture of the response was taken for later analyses. Participants were then required to remove their index finger (right or left) and place it back on the table, before the next landmark was read. Participants did not receive any feedback during the experiment.

#### Data processing

Information on the coordinates for single pointing responses was used to calculate the misallocation judgements of each landmark. Thus, from each individual picture taken, the *x* and *y* coordinates were calculated for the real and perceived location per landmark (origin was located at the bottom right corner of each picture). For this, a programme developed with Borland C^++^ Builder (2007) was used, allowing conversion of pixel units into centimetres.

The coordinate data were further used to calculate the inferred hand size (lengths and widths). Previous studies have used the information on the coordinates for single pointing responses to calculate distance between landmarks, and decode the so-called body model (e.g., Longo and Haggard 2012). These distances are chosen between meaningful pairs of landmarks to calculate the length of fingers and the width of the hand (e.g., between H2 and H3 for the length of the thumb; see Fig. 1b). Thus, the finger lengths, the hand’s dorsum length, the hand’s width, and the width of the wrist were calculated for each hand in near and far conditions. From raw length and width data, a percentage of distortion was calculated as per previous studies (e.g., Longo and Haggard 2010), with the following formula: [(perceived size-real size)/real size] × 100. This calculation provided the percentage of distortion (i.e., overestimation or underestimation).

#### Statistical analyses

The results on the representation of the hands are considered in Condition 1 (near-reaching space), and again in Condition 2 (far-reaching space). First, the overall distortion for each condition was calculated and tested against zero (no distortion) for each group. Differences between groups were tested by means of independent two-tailed *t *tests for each dimension (finger lengths, dorsum lengths, hand width, and wrist width). Pairwise *t* tests were used to test differences in the representation of different dimensions within participant groups (e.g., difference in hand width versus wrist width). Alpha level was set at *p* > 0.05. Effect size was estimated using Cohen’s *d* calculation. Finally, a set of 2 (Group: SL interpreters and controls) by 2 (Distance: near-reaching and far-reaching space) mixed-model ANOVAs were run to compare size distortion between conditions for each dependent variable (i.e., finger length, dorsum length, hand width, and wrist width). Alpha level was again set at *p* > 0.05 and partial eta square (*η*_p_^2^) values were calculated for the effect size.

### Results

**Fig. 2:**
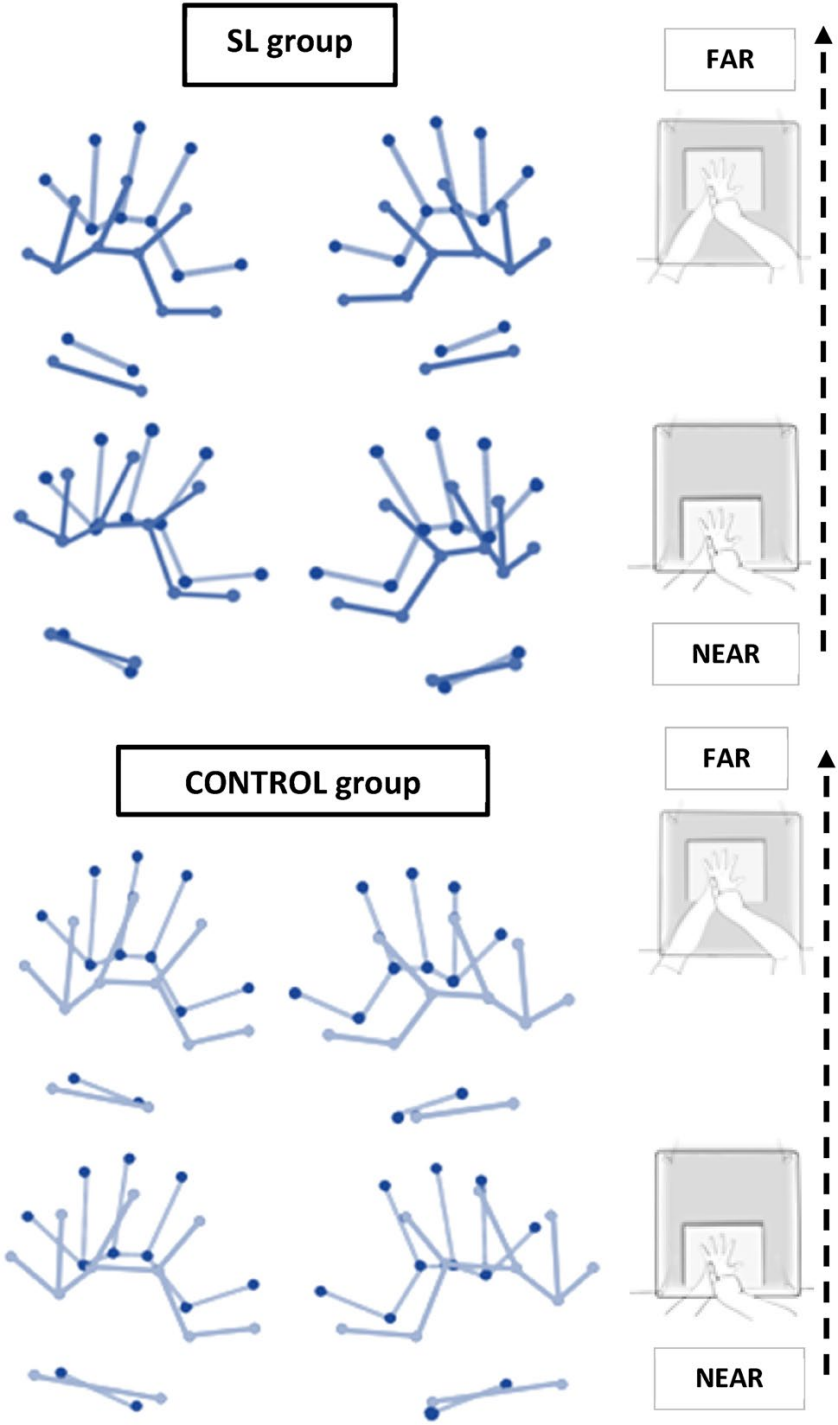
Cartographic hand maps. Maps for real (lighter lines with darker dots in the background) and perceived hands’ representation in SL (darker lines in the front) and Control (lighter lines in the front) groups, in near and far conditions

Maps of the real and perceived hands for both groups were produced using the *x* and *y* coordinates, showing the differences between the real and perceived hand sizes between conditions and groups (see Fig. 2).

#### Condition 1: near-reaching space

Differences between left- and right-hand size estimations were not significant (all *p*s. > 0.05 for both groups and lengths); hence, size (percentage of over/underestimation) was averaged across hands for further analyses.

There was an overall underestimation of finger lengths, with SL participants underestimating their length by a − 21.23% (SD = 11.24) and controls by a − 12.53% (SD = 14.11). The distortion of length was significant for both SL [*t*(9) = − 5.97, *p* < 0.001, *d* = − 1.88] and controls [*t*(9) = − 2.82, *p* = 0.02, *d* = − 0.88]. Differences between groups did not reach significance [*t*(18) = 1.53, *p* = 0.15, *d* = 0.68].

The length of the hands’ dorsum was calculated as the distance between the second interspace (H5) and the interior part of the wrist (H1) (see Fig. 1b). Overall, the perceived length of the dorsum was underestimated in both groups. SL group underestimated the size of the dorsum by − 9.96% (SD = 13.1) and the distortion was significant [*t*(9) = − 2.4, *p* = 0.04, *d* = 0.76]. Controls underestimated in similar magnitude (M = − 9.56%, SD = 21.49) but in this case not significantly so [*t*(9) = − 1.41, *p* = 0.19, *d* = 0.44]. Differences between groups did not reach significance [*t*(18) = 0.05, *p* = 0.96, *d* = 0.02]. These results taken together confirm a similar general pattern of distortions in length perception between groups in the near condition. To compare if the length distortion was different between fingers and the dorsum, a pairwise *t* test was run within each group (Bonferroni-corrected *p* value of *p* = 0.025). Interestingly, there were significant differences in the SL group [*t*(9) = − 3.75, *p* = 0.005, *d* = 1.19], whereas no differences were found for controls [*t*(9) = − 0.46, *p* = 0.66, *d* = 0.14].

**Fig. 3:**
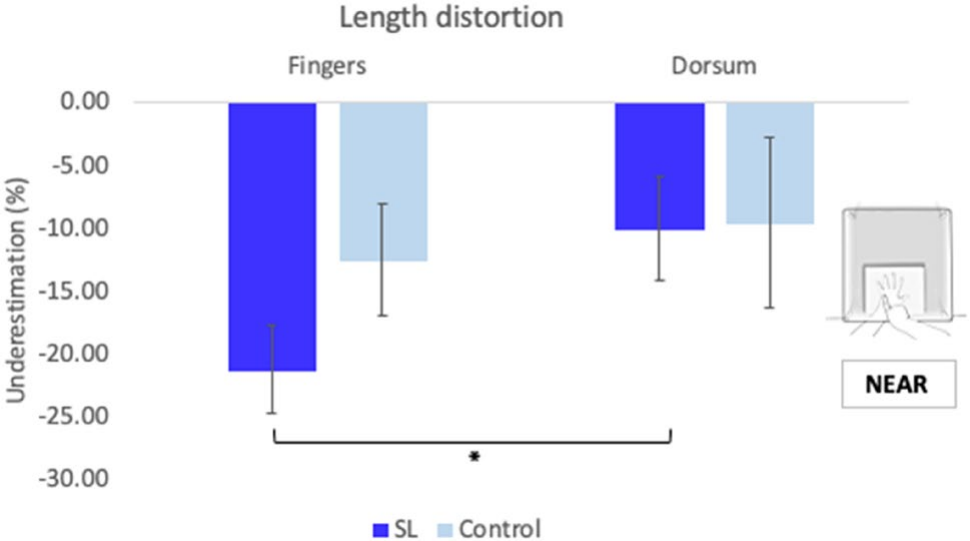
Length distortion near reaching space. Averaged underestimation for finger lengths and length of hand’s dorsum across hands for controls and SL participants. SL participants significantly underestimated the length of their fingers more than their hands’ dorsum. Error bars represent Standard Error of the Mean. * Denotes significant differences

These results indicate a different pattern of distortions within groups, with the length of fingers in the SL group more underestimated than the dorsum (see Fig. 3).

**Fig. 4:**
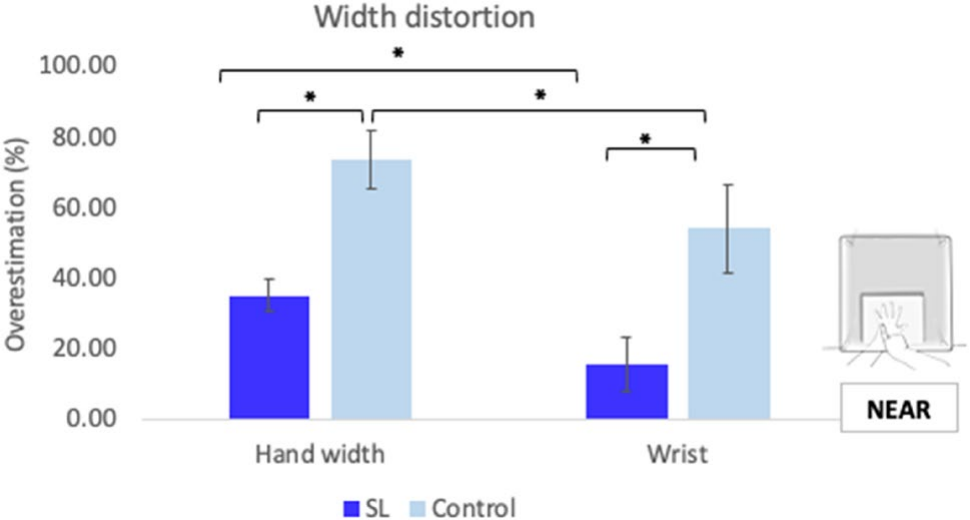
Width distortion in near-reaching space. Representation of the distortion of hands’ and wrists’ widths averaged across hands for both groups. SL interpreters showed reduced distortion for hands and wrists widths. The distortion of hand width was significantly larger in both groups. Error bars represent the Standard Error of the Mean. * Denote significant differences

The real and perceived distance between second (H5) and fourth interspace (H9) was calculated as a measure of overall width of the hands (see Fig. 1b). Both groups overestimated the width of their hands, with SL users (M = 35.09%, SD = 14.56); [*t*(9) = 7.62, *p* < 0.001, *d* = 2.41] and controls (M = 73.55%, SD = 26.16); [*t*(9) = 8.89, *p* < 0.001, *d* = 2.81] showing a significant distortion. The difference between groups was significant [*t*(18) = 4.06, *p* = 0.001, *d* = 1.82], confirming that the SL group was more accurate than controls in the representation of the width of their hands (see Fig. 4).

To explore the effect on width representation further, we considered the width of the wrists (see Fig. 4). SL participants did not show a significant overestimation of their width (M = 15.65%, SD = 24.65); [*t*(9) = 2.01, *p* = 0.08, *d* = 0.63]. In contrast, controls perceived their wrists to be wider than their real size (M = 54%, SD = 39.27); [*t*(9) = 4.35, *p* = 0.002, *d* = 1.37]. Group differences were significant [*t*(18) = 2.62, *p* = 0.018, *d* = 1.17], confirming that the SL participants were more accurate when estimating the size of their wrists. As with lengths, the perceived width distortion was compared between hands and wrists (Bonferroni-corrected p value of 0.025). There was a trend in the SL group [*t*(9) = 2.36, *p* = 0.04, *d* = 0.75], indicating a more distorted representation of the hand in comparison with the wrist. This was also the case for controls, who also perceived the hand significantly more distorted than the wrist [*t*(9) = 2.74, *p* = 0.02, *d* = 0.86]. Hence, it appears that the hand width overestimation is more accentuated in the hand than the wrist in both groups.

#### Condition 2: far-reaching space

**Fig. 5:**
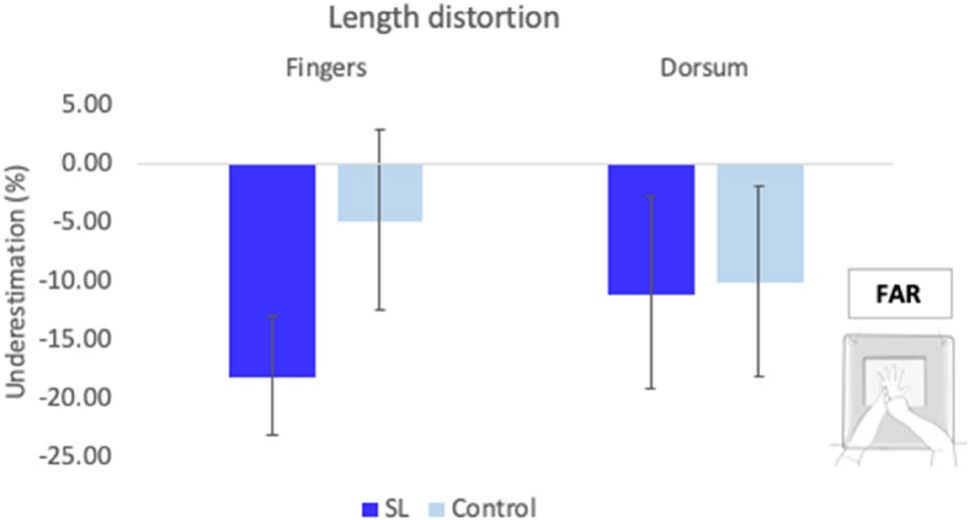
Length distortion far-reaching condition. Representation of the averaged distortion for the length of fingers and the length of the hand’s dorsum averaged across hands for both groups. There were no significant differences between groups, which similarly underestimated their lengths of fingers and dorsum. Error bars represent the Standard Error of the Mean

In the far distance, SL participants significantly underestimated the length of fingers (M = − 17.99%, SD = 15.74); [*t*(9) = − 3.62, *p* = 0.006, *d* = − 1.14], whilst controls did not show a significant distortion of their finger length (M = 4.64%, SD = 21.09); [*t*(9) = − 0.696, *p* = 0.5, *d* = − 0.22] (see Fig. 5). However, differences between groups were not significant [*t*(18) = 1.61, *p* = 0.13, *d* = 0.72].

When considering hands dorsum’s lengths, SL participants underestimated by − 10.91% (SD = 22.09), but the distortion was not significant [*t*(9) = − 1.56, *p* = 0.15, *d* = 0.49]. Similarly, controls showed underestimation of the size of the dorsum (M = − 9.91%, SD = 26.5) but not significantly so [*t*(9) = − 1.18, *p* = 0.27, *d* = 0.37]. Differences between groups did not reach significance [*t*(18) = 0.09, *p* = 0.93, *d* = 0.04]. Differences between the underestimation of finger lengths and dorsum were not significant for either the SL group [*t*(9) = − 0.98, *p* = 0.35, *d* = 0.31] or the control group [*t*(9) = 0.52, *p* = 0.61, *d* = 0.17].

**Fig. 6:**
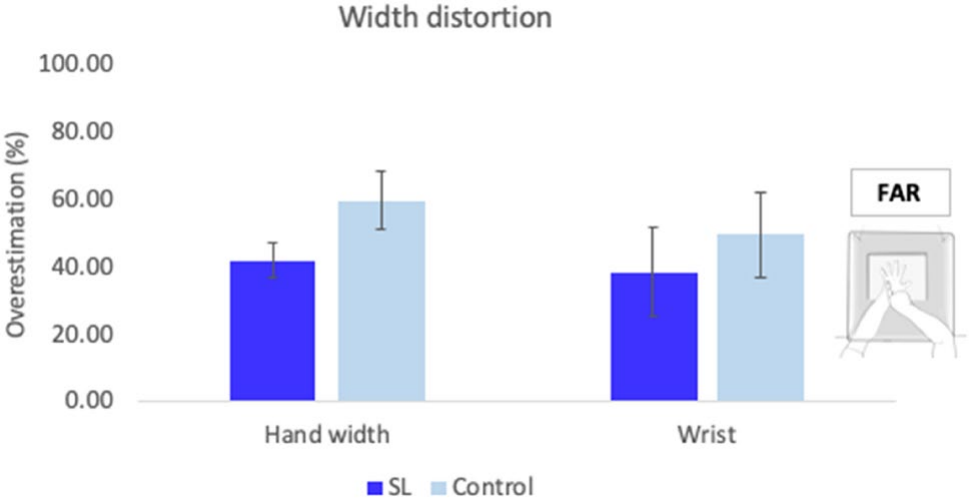
Width distortion far reaching space condition. Representation of the averaged width of hands and wrists for both groups. There were no significant differences between groups in the distortion of the width of the hands and the wrists. Error bars represent the Standard Error of the Mean

In the far distance, SL participants perceived the width of their hands significantly overestimated in size (M = 41.93%, SD = 16.55); [*t*(9) = 8.01, *p* < 0.001, *d* = 2.53]. Similarly, controls also showed a distortion for the width of their hands, and was found to be significant (M = 59.81%, SD = 27.13); [*t*(9) = 6.97, *p* < 0.001, *d* = 2.21] (see Fig. 6). Differences between groups were not significant [*t*(18) = 1.78, *p* = 0.09, *d* = 0.8].

Similarly, overestimation of width was present for the wrists in both the SL group (M = 38.56%, SD = 42.2); [*t*(9) = 2.89, *p* = 0.02, *d* = 0.91] and the control group (M = 49.68%, SD = 39.66); [*t*(9) = 3.96, *p* = 0.003, *d* = 1.25]. As with the hands, differences between groups did not reach significance [*t*(18) = 0.61, *p* = 0.55, *d* = 0.27], and are in contrast with the near space condition. Finally, differences between the perceived width of the hands and wrists were not significant in either the SL [*t*(9) = 0.26, *p* = 0.8, *d* = 0.08] or the control groups [*t*(9) = 0.86, *p* = 0.41, *d* = 0.27], confirming that hands and wrists were equally distorted in far-reaching space condition.

A summary table of the results of all the analyses in previous sections can be found in Table 2.

**Table 2:** Summary table with statistical analyses for the hand representation for both groups and both conditions

Location	Body part	Group	Mean distortion in % (SD)	*p* values (one-sample *t* test)	*p* values (independent sample *t* test)	Further comparisons		*p* values (pairwise comparisons)
Near-reaching space	Finger lengths (%)	SL	− 21.23% (11.24)	**< 0.001**	0.15	Between finger and dorsum lengths	SL	**0.005**
		Controls	− 12.53% (14.11)	**0.02**				
	Dorsum length (%)	SL	− 9.96% (13.1)	**0.04**	0.96		Controls	0.66
		Controls	− 9.56% (21.49)	0.19				
	Hand width (%)	SL	35.09% (14.56)	**< 0.001**	**0.001**	Between hand and wrist widths	SL	**0.0**4
		Controls	73.55% (26.16)	**< 0.001**				
	Wrist width (%)	SL	15.56% (24.65)	0.08	**0.018**		Controls	**0.02**
		Controls	54% (39.27)	**0.002**				
Far-reaching space	Finger lengths (%)	SL	− 17.99% (15.74)	**0.006**	0.13	Between finger and dorsum lengths	SL	0.35
		Controls	4.64% (21.09)	0.5				
	Dorsum length (%)	SL	− 10.91% (22.09)	0.15	0.93		Controls	0.61
		Controls	− 9.91% (26.5)	0.27				
	Hand width (%)	SL	41.93% (16.55)	**< 0.001**	0.09	Between hand and wrist widths	SL	0.8
		Controls	59.81% (27.13)	**< 0.001**				
	Wrist width (%)	SL	38.56% (42.2)	**0.02**	0.55		Controls	0.41
		Controls	49.68% (39.66)	**0.003**				

Percentage of over/underestimation (with standard deviation) is provided for all areas and groups (SL and controls) and conditions (near-reaching and far-reaching space). Significance values are included for all comparisons. A significant *p* value is marked in bold

#### Comparisons between conditions

**Fig. 7:**
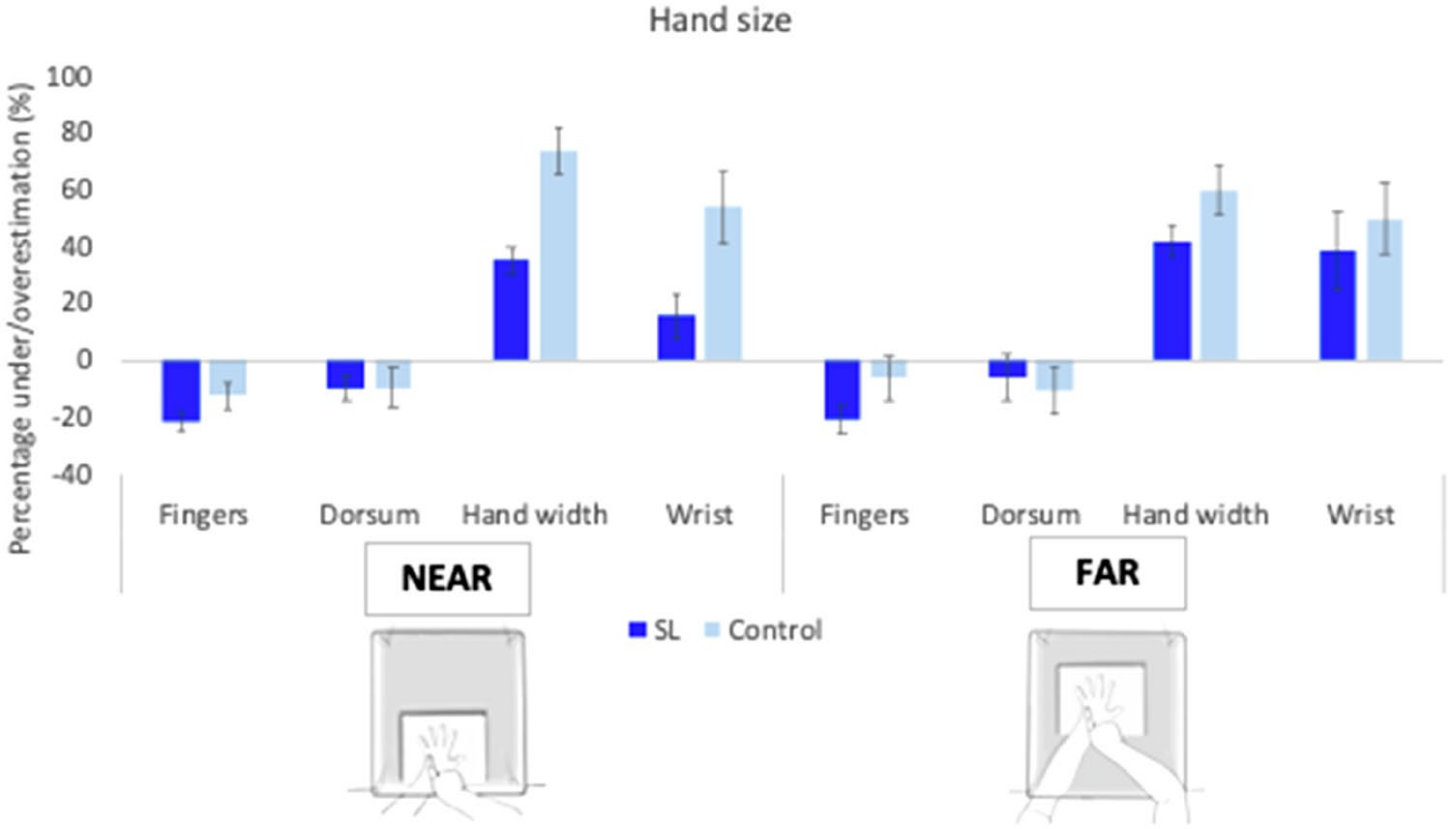
Comparisons between conditions. Averaged size distortion for finger lengths, hands’ dorsum, hands’ width and wrists for all participants across conditions. Error bars indicate the Standard Error of the Mean

In this case, the differences found in the perception of the size of the hand and wrist across distance conditions were directly compared. Mixed model ANOVAs were run with two factors: Distance (near and far) and Group (control and SL groups), for each dependent variable (finger lengths, dorsum length, hand width, and width of wrists). Averaged results are presented in Fig. 7.

The ANOVA for the length of fingers showed a trend for the Distance factor [*F*(1,18) = 4.01, *p* = 0.06, *η*_p_^2^ = 0.18], as there was an overall reduction of the underestimation in the far-reaching space condition. Results did not reach significance for the Group factor [*F*(1,18) = 2.82, *p* = 0.11, *η*_p_^2^ = 0.14], or for the Distance-by-Group interaction [*F*(1,18) = 0.7, *p* = 0.41, *η*_p_^2^ = 0.04].

For the dorsum’s length, neither Distance [*F*(1,18) = 0.02, *p* = 0.89, *η*_p_^2^ = 0.001], Group [*F*(1,18) = 0.01, *p* = 0.93, *η*_p_^2^ < 0.001], nor Distance-by-Group interaction [*F*(1,18) = 0.004, *p* = 0.95, *η*_p_^2^ < 0.001] were significant.

When considering the hand width, the ANOVA did not reveal significant results for the main factor of Distance [*F*(1,18) = 0.39, *p* = 0.54, *η*_p_^2^ = 0.02]. However, there were significant results for the main effect of Group [*F*(1,18) = 12.27, *p* = 0.003, *η*_p_^2^ = 0.41], confirming a better overall estimation of hand width in the SL group. Finally, there was a trend for the Distance-by-Group interaction [*F*(1,18) = 3.46, *p* = 0.08, *η*_p_^2^ = 0.16], as the SL group perceived the width of their hand less distorted in the near space condition.

Finally, the ANOVA for the wrist did not reveal significant results for Distance [*F*(1,18) = 1.33, *p* = 0.26, η*p*² = 0.07], Group [*F*(1,18) = 2.91, *p* = 0.11, η*p*² = 0.14], or for the Distance-by-Group interaction [*F*(1,18) = 2.85, *p* = 0.11, η*p*² = 0.14].

### Preliminary discussion

Results in this study have shown how expert signing modulates the representation of hands. Specifically, distortions in near-reaching space follow the characteristic pattern for both groups (i.e., underestimation of length and overestimation of width), with SL group showing a significant reduction of distortion for width. SL interpreters were not better than the control group when estimating their finger lengths. These results are in line with Coelho et al.’s (2019) study, in which a smaller hand was seen as advantageous to catching the ball in baseball, reducing the margin of error. Extrapolating to SL users, it is possible that the way the hands are used also affects how these are represented. To sign, hands need to be moved accurately, and in a quick and timely fashion, coordinating complex movements. Therefore, it could be argued that a smaller hand representation may be of more benefit, in the same way that certainty reduces the size of hand aperture for grasping (Jakobson and Goodale 1991).

Regarding the specific direction of the distortions, SL and control groups did not significantly differ in the perception of finger lengths and did not reflect any clear effects of expertise. However, within the SL group, worse performance (i.e., more distortion) was demonstrated at the estimation of finger length compared to that of the dorsum. This confirmed the association of body size representation with specific use and functional experience (Caggiano and Cocchini 2020; Ferretti 2016; Fraser and Harris 2016, 2017; Romano et al. 2019), and not with an overall bias to underestimating lengths. In other words, the larger underestimation of length was specific for fingers, and not a general tendency affecting the whole hand. Repeated skill work in a given manual workspace will prime the perception of hand position towards usual locations, biasing localisation towards them (Fraser and Harris 2016). In this case, SL practitioners vary the position of their fingers frequently, perhaps increasing the uncertainty of their localisation. Hence, functionality becomes a main factor that guides proprioceptive localisation of fingers (Dandu et al. 2018). Furthermore, the specific distortions directly depend on the perceptual experience with the body part (Bettger et al. 1997). Supporting this, other studies have postulated that dancers are only better in the localisation of highly trained postures, which does not necessarily transfer to non-trained postures (Jola et al. 2011; Schmitt et al. 2005).

On the contrary, SL participants showed a clear advantage in the representation of the width of hands and wrists. Width is believed to be the dimension with more variability, as it is intrinsically related to more representational flexibility to accommodate growth (De Vignemont et al. 2005; Hashimoto and Iriki 2013). Moreover, width is the dimension that appears more linked to own body representation (Ganea and Longo 2017), as length underestimation is also found when judging the size of a rubber hand (Longo et al. 2015; Saulton et al. 2016). Hence, width appears more susceptible to modulation than length. In any case, it follows that expert use of the hands modulates influences in width perception [e.g., homuncular characteristics (Nguyen et al. 2005); reversed distortion (Linkenauger et al. 2015); self-perception biases (Felisberti and Musholt 2014; Sui and Humphreys 2015), and safety margin (Nico et al. 2010)], in such a way that it becomes more accurate.

Owing to the idea of manual practice and functional workspace (Fraser and Harris 2016, 2017), differences in the representation of the hands were only seen in the near-reaching space, within the boundaries of the signing space, and not in the far-reaching space, whereby performance between groups was not significantly different. This was due to a reduction of the gain by the SL group in near-reaching space from 38.46 to 17.88% in far-reaching space. As signers produce all their communication within a confined three-dimensional signing space that mostly extends from the forehead to the waist, to the front of the face and chest, and laterally beyond the elbows (Arnold and Mills 2001; Emmorey 2001), differences in experience may only be found within this confined space. In particular, BSL signs occur near the other hand, face, or trunk (Woodward 1982), and the categorisation of handshapes in space is important to provide meaning (Cormier et al. 2015; Sandler 2018). Therefore, the effect of SL expertise on hand proprioception was limited to the space experts normally sign in, as represented by the near-reaching space condition.

Not only do SL practitioners use the hands to a greater extent than the typical population, but they also sign around the face, as well as using facial expressions to communicate (Emmorey and McCullough 2009). Thus, the next experiment looked into the effect of expertise for the metric representation of the face.

## Experiment 2

Head tilts, movements of the brows, squinting of eyes, and mouth movements are used independent of the hands in SL, and each component provides meaning in different ways. That is, upper face areas, such as brows or eyes, when combined with hand gestures provide intonation to the expressions (Sandler 2018). In contrast, the lower face areas are involved in different functions. Mouthings are speech-like mouth movements and have phonological function; whilst mouth gestures are non-speech-like movements that are inseparable, and guided by the manual action not deriving from words (Capek et al. 2008).

SL proficient users focus on the addressee’s face, and seldom look to their hands (Capek et al. 2008; Siple 1978). Similarly, the addressee focuses on the signer’s face, where gestures are seen in high acuity (foveal vision), which becomes the centre of attention (Muir and Richardson 2005). In contrast, eye fixations to manual gestures are minimal and only present when gestures occur near the face, otherwise being processed by peripheral vision (Muir and Richardson 2005; Siple 1978). The specialised use of the face in SL not only translates in attentional differences, but also on improved perceptual abilities. For example, SL proficiency is associated with enhanced lip-reading skills, in particular for deaf people (MacSweeney et al. 2008), and improved local facial feature recognition (Emmorey and McCullough 2009). In fact, better discrimination of self-face is linked to a more robust stored representation (Keyes and Brady 2010). These findings support the importance of the face in SL, and the relevance of studying the effects of expertise on its representation. In this second study, improved ability of SL practitioners to localise face landmarks is predicted in comparison with controls.

### Methods and procedures

#### Participants

The same group of participants took part in this second experiment. See demographic information in Table 1. Experiment 2 took place on the same day as the previous one, and the order was counterbalanced across participants to control from order or practice effects. No feedback was provided after Experiment 1, and participants were engaged in general conversation before carrying out Experiment 2 (or vice versa).

#### Face localisation task and procedure

**Fig. 8:**
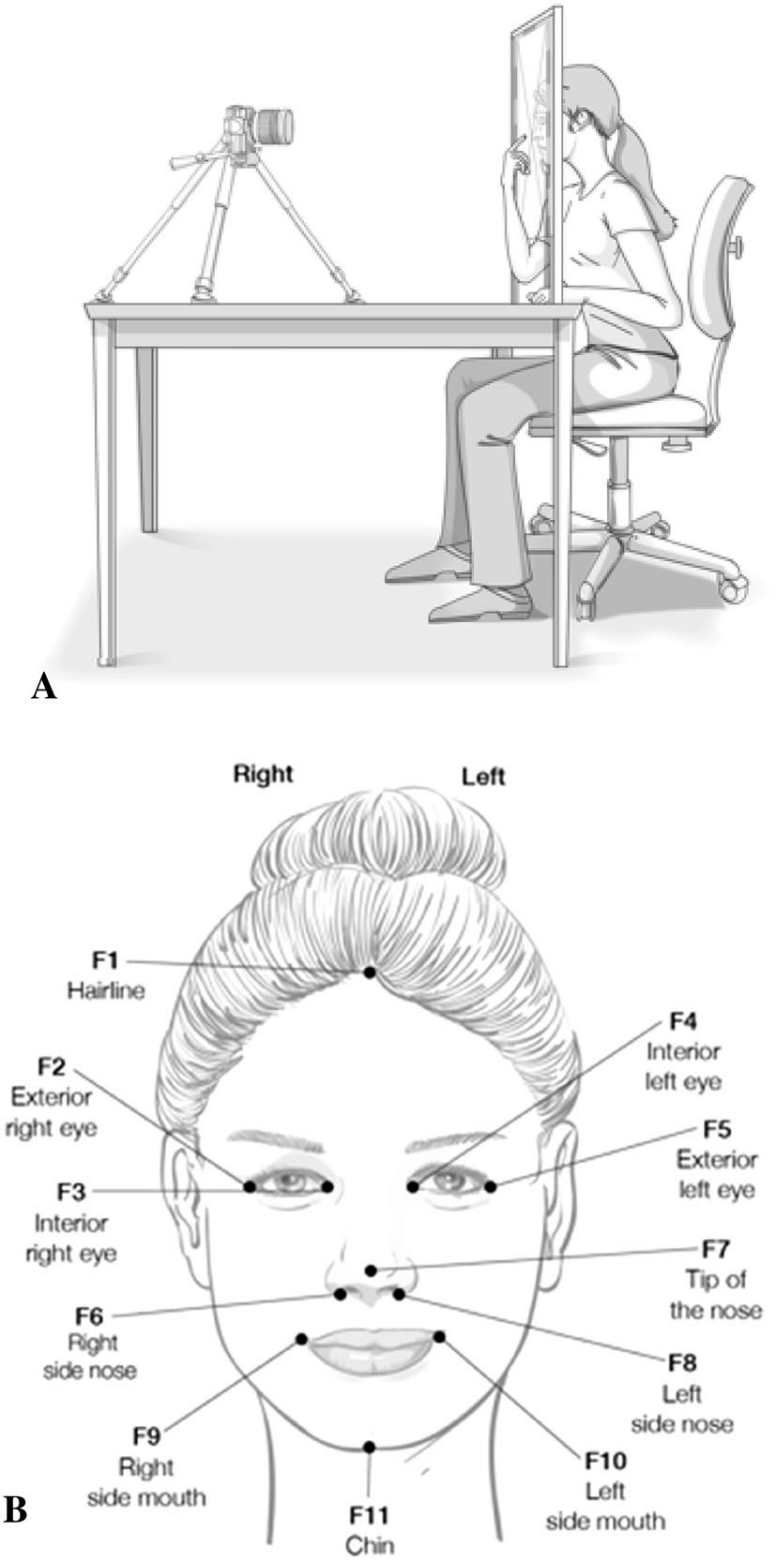
Face localisation task. Face apparatus (**a**) and illustration of face landmarks (**b**)

Similar to previous studies on face metric representation (Longo and Holmes 2020; Mora et al. 2018), participants were required to locate different face landmarks on command whilst keeping their eyes closed. A vertical transparent Perspex board (50 × 55 cm) resting on two wooden legs (20 cm height) was positioned on a table, in front of the participant. A chin rest was on the edge of the table, between the Perspex board and the participant. The Nikon 3200D camera was positioned on a tripod at 120 cm. The centre focus of the camera was aligned with the centre of the board, and the tape measures were attached to the top and right sides of the board for later analyses (see Fig. 8a).

Participants were required to rest their head on the chin rest, aligning the tip of the nose with the camera focus. They were asked to avoid movements of the face during the whole duration of the experiment, to maintain a relaxed face expression (not smiling), and to keep their eyes closed. As in Mora et al. (2018), 11 landmarks were read aloud in random order (see Fig. 8b). The landmarks had to be located by pointing towards the face, on top of the Perspex board, with their right index finger. A picture (6016 × 4000 pixels) was taken for each pointing response. Following this, the hand had to return to the initial position on the table, before the next landmark was requested. Each landmark was repeated twice, with a total of 22 trials per participant. Participants did not receive any feedback for the whole duration of the experiment. As in Experiment 1, participants practiced identifying the landmarks on a schematic drawing prior the experiment.

#### Data processing

Pictures were analysed using Borland C^++^ Builder (2007). A total of 22 pictures (2 for each of the 11 landmarks) were collected. Pixel units were converted into centimetres, to obtain the *x* and *y* coordinates for each real and perceived landmark location. The origin in this case was at the left top corner of each picture. The real and perceived distances between landmarks was then calculated for specific areas. The length of the face was calculated by obtaining the real and perceived distances between the F1 and F11 landmarks. Further to this, the distance between different facial features were calculated: right eye (distance from F2 to F3); between eyes (distance between F3 and F4); left eye (distance from F4–F5); nose (F6 to F8); and mouth (F9–F10) (see Fig. 8b). Percentage of over/underestimation was calculated from these data.

One participant (SL003) had a missing data point for the right eye. We replaced the missing data with the series mean for the purposes of conducting the analyses.

#### Statistical analyses

As in the previous experiment, initial one-sample t tests were run to investigate if the distortions of size were significantly different from zero (no distortion). Group differences were then investigated by means of mixed-model ANOVAs or independent *t* tests. Alpha level was set at *p* > 0.05. Partial eta square (*η*_p_^2^) for ANOVAs and Cohen’s *d* for *t *tests were calculated for the effect size.

### Results

**Fig. 9:**
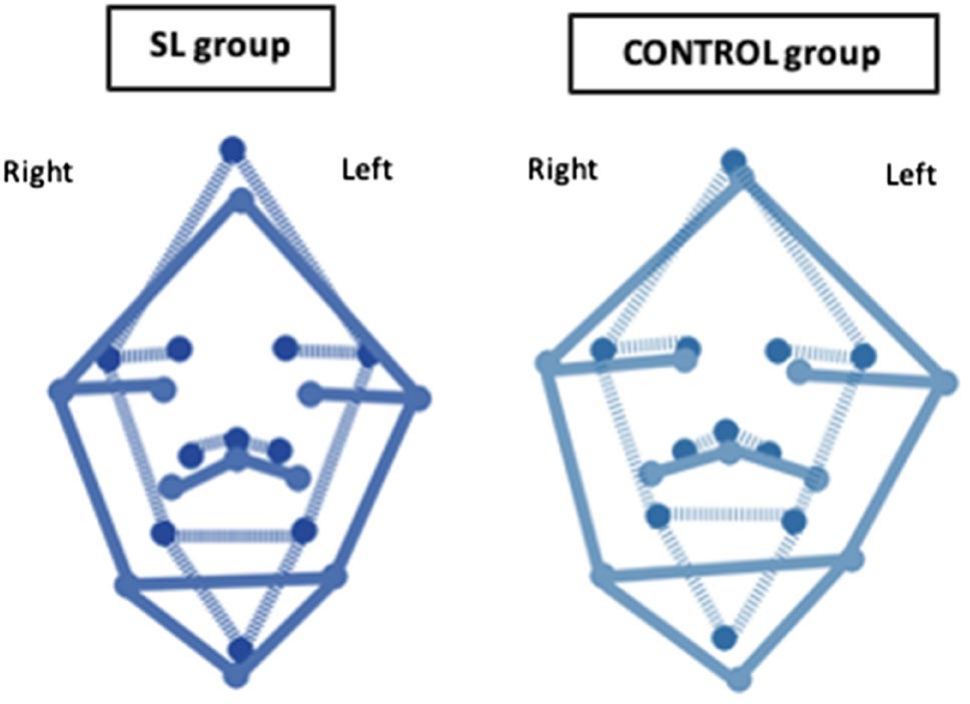
Face maps. Representation of real (continuous lines) and perceived face size (dotted lines) in SL and control groups

The coordinates were used to produce schematic maps of the real and perceived face for both groups (see Fig. 9).

**Fig. 10:**
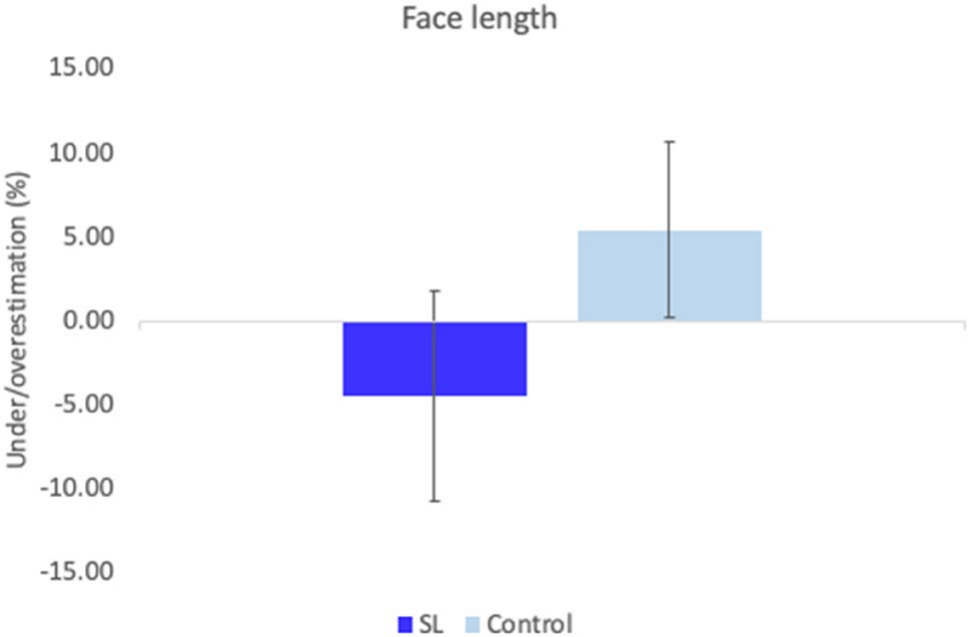
Face length. Representation of the length of the face for both SL and controls. There were no significant differences between groups in the perceived length of the face. Error bars represent the Standard Error of the Mean

SL and control participants showed opposite trends in length perception, but the distortion was not significant (i.e., different from zero) for either the SL (M = − 4.5%, SD = 19.87); [*t*(9) = − 0.72, *p* = 0.49, *d* = − 0.23] or control groups (M = 5.39%, SD = 16.51); [*t*(9) = 1.03, *p* = 0.33, *d* = 0.33]. An independent samples *t* test confirmed these differences were not significant between groups [*t*(18) = 1.21, *p* = 0.24, *d* = 0.54] (see Fig. 10). On average, the SL group showed more accuracy in the perception of the width of the face. The control group overestimated the width of face landmarks by 73.76% (SD = 19.64) a distortion that was significant [*t*(9) = 11.87, *p* < 0.001, *d* = 3.75]; whereas the SL group overestimated by 36.55% (SD = 19.92), again, significantly so [*t*(9) = 6.78, *p* < 0.001, *d* = 2.14].

**Fig. 11:**
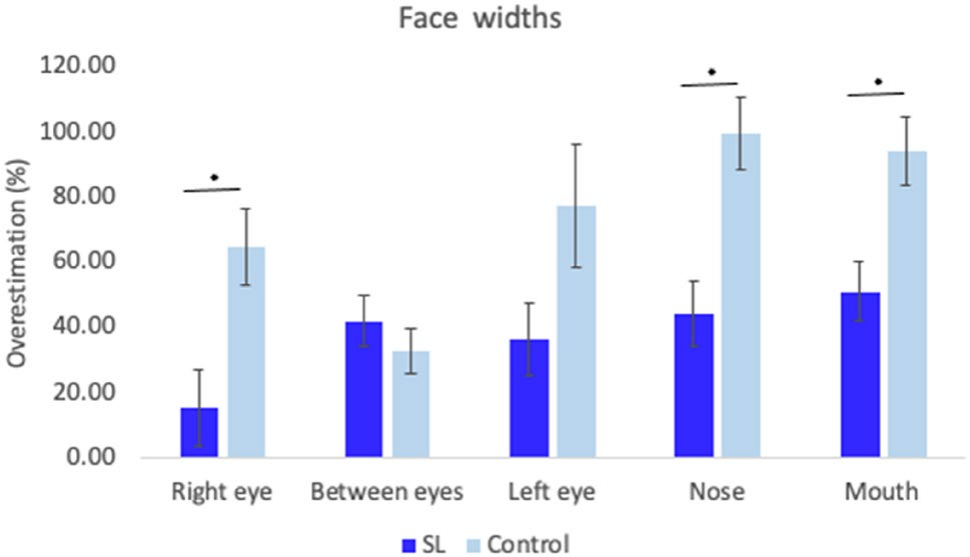
Face width distortion. Graph depicting representation of the width of face landmarks for both groups. Error bars represent the Standard Error of the Mean. * Denote significant differences. SL group perceived the right eye, the nose and the mouth less distorted than controls

Figure 11 illustrates the width sizes for each face Landmark. A mixed-model ANOVA (Landmarks (5) × Group (2)) revealed a significant main effect of Landmarks [*F*(4, 72) = 4.74, *p* = 0.002, *η*_p_^2^ = 0.21], indicating different width size representation depending on the landmark considered. Bonferroni corrected pairwise comparisons (cut off *p* value of 0.005), showed that the mouth was more overestimated than the right eye (*p* = 0.003; mean difference = 31.81) and the between eyes area (*p* = 0.003; mean difference 34.46). It also showed that the nose width was more overestimated than the between eyes area (*p* = 0.003; mean difference = 34.36). No other comparisons reached significance (all *p*s. > 0.005). Furthermore, there was a main effect of Group [*F*(1,18) = 19.02, *p* < 0.001, *η*_p_^2^ = 0.51], confirming that SL participants represented the width of the face more accurately than controls (mean difference = 36.12). Finally, the Landmarks-by-Group interaction was significant [*F*(4,72) = 2.86, *p* = 0.03, *η*_p_^2^ = 0.14], with different distortion of landmarks depending on the group considered. Independent Bonferroni-corrected *t *tests (*p* value of 0.01) revealed that SL participants perceived the right eye [*t*(18) = 2.99, *p* = 0.008, *d* = 1.34], the nose [*t*(18) = 3.74, *p* = 0.001, *d* = 1.67], and the mouth [*t*(18) = 3.19, *p* = 0.005, *d* = 1.43], significantly more accurately than controls. There were no significant differences for the left eye (*p* = 0.08) or between eyes area (*p* = 0.4).

## General discussion

Previous studies have shown that metric distortions of hands and faces are intrinsic to healthy representation of the body and can be modulated by intensive long-term training (Cocchini et al. 2018; Coelho et al. 2019; Romano et al. 2019). Despite the interest on the representation of hands (see Longo 2017, 2019 for a recent review), few studies have looked at the impact of extensive tool use on hand representation (e.g., Cocchini et al. 2018; Coelho et al. 2019), and none have explored the modulation of the representation and interaction with space localisation. Furthermore, no other studies had looked into the malleability of the representation of the face. Therefore, SL expert users were considered to explore the representations of both body areas and links to space of manual action, due to their particular training and expertise.

In Experiment 1, SL experts considerably outperformed controls at estimating the width of their hands in near-reaching space, but not in far. These results confirmed the link to functional workspace and size representation (Fraser and Harris 2016). Hence, expertise does not modify the mental representation of the hands disregarding localisation; instead, it is intrinsically linked to space. Interestingly, this advantage in the representation was specific of the width, not of the lengths. These results contrast with findings in a previous study with magicians (Cocchini et al. 2018), in which their expertise was associated with improved finger length perception. This difference may be due to the type of expertise. Magicians are experts on the instrumental use of hands; that is, they have trained to use them when holding objects, improving their sleight of hands and, in particular, refining the representation of their fingers, which are highly trained (Cavina-Pratesi et al. 2011). Instead, SL is an embodied visual-spatial language and practitioners use hands and face for language and communication (Shield and Meier 2018), but do not train the sleight of hand to manipulate certain objects or ‘deceive’, as in the case with magicians. The importance of the use of hands in SL includes which handshape they adopt, where they are located in relation to other body parts, and in which direction they are moving (Mitchell et al. 2013; Sehyr and Cormier 2016). Moreover, the link between better representation and expertise may not be as straight forward. Instead, these results may indicate that the body distortions may be modulated in the direction that best fits each type of expertise (Coelho et al. 2019). On some occasions, expertise does improve representation, as seen in magicians (Cocchini et al. 2018) and in the width representation in SL practitioners, whereas at other times, it does not, as seen in finger length perception in SL experts and in baseball players (Coelho et al. 2019). Similarly, gains in proprioceptive localisation of body segments are only seen for highly trained postures in dancers, but these gains are not generalised to non-trained ones (Jola et al. 2011; Schmitt et al. 2005). Furthermore, a recent study with SL practitioners did not find improved performance in body imagery-related tasks, confirming that body representation may be affected differently depending on the tasks considered (Brusa et al. 2021). This type of hand use may change the representation in comparison with controls, but may not necessarily cause an overall improved representation; rather, evidence suggests that it modulates representation in the direction that is linked to the function in hand. Hence, the effect of expertise may not be the same for other body parts, such as the face.

In the second experiment, the metric representation of the face was explored. In this case, SL users perceived the width of face features more accurately than controls, whereas no differences were found in length perception. Hence, it is also the case that the representation of the face tends to be smaller for SL, which is, overall, more accurate. These differences with hands representations may be due to the differences in their mobility and use. Indeed, hands can change position and shape in more degree than the face. Moreover, SL practitioners experience these body parts in different ways. They do not visually track the movement of their hands when signing, and vision is instead used to calibrate the signing space, relying on somatosensory, kinaesthetic, and tactile feedback (Emmorey et al. 2009a, b), as in magicians (Cavina-Pratesi et al. 2011). They look at the face of the addressee (Emmorey et al. 2009a, b; Siple 1978), and signs fall in the periphery or outside of the visual field (Emmorey et al. 2009a, b). Instead, SL experts show superior face recognition skills, directly linked to the expertise in signing (Bettger et al. 1997). In particular, expertise with SL fine-tunes face-processing skills, such as local facial features discrimination (Emmorey and McCullough 2009), rather than just enhancing overall visual discrimination (McCullough and Emmorey 1997). For example, studies have shown processing skills that are particularly strong when identifying subtle facial feature changes in eye configuration or mouth shape (McCullough and Emmorey 1997). This was associated with the experience with SL and lip-reading skills (McCullough and Emmorey 1997), and not with the experience of deafness (Parasnis et al. 1996). Interestingly, this advantage disappears with inverted faces, in which signers perform as non-signers, confirming the gain directly depends on the perceptual experience with the body part (Bettger et al. 1997). Furthermore, attention to faces in the general population is directed to the upper areas/eyes, whilst in the case of signers, there is an equal distribution to upper and lower areas (Letourneau and Mitchell 2011; Mitchell 2017), with a preference or salience for lower ones (Mitchell et al. 2013). This may explain the general improvement in the representation of all face features. Furthermore, this highly developed skill in face processing will help construct a more robust self-face representation, with greater detailed information on spacing between features, instrumental for own face discrimination (Tsao and Livingstone 2008). Finally, the improved face representation may also be linked to usual workspace, as the face localisation task was also circumscribed to the space in which signs occur, as in near-reaching space for the hands.

The size distortions found in the second experiment support the previous studies that reported overestimation of width perception for the face, consistently found with a variety of methods (D’Amour and Harris 2017; Fuentes et al. 2013; Mora et al. 2018). As with hands, it is the width element the one more distorted, and where differences are found between groups. In general, the perception of the body seems to be overestimated in width consistently across body parts and groups (e.g., Longo 2017). Moreover, studies showing variability on body size perception have observed differences in this dimension, probably to accommodate growth (De Vignemont et al. 2005), as height/length is rather stable in adulthood (Hashimoto and Iriki 2013). Thus, it is not surprising that modulation of representation due to expertise may affect this dimension. In fact, studies in embodiment of body parts show a preference of the brain to embody larger body parts (Haggard and Jundi 2009; Pavani and Zampini 2007), and effects in grasping are seen after enlarging the hand, not when shrinking it (Marino et al. 2010). Furthermore, experiments have also shown a preference for enlarged hands in the fake-hand illusion (Pavani and Zampini 2007). Thus, width dimension may be associated with more variability, thus being more susceptible to the effects of long-term expertise.

Finally, attentional components cannot be completely disregarded from the improved performance by SL practitioners in the hand task. Similarly to the face, it may be that more attention focused to the hands in near space is linked to the improved representation of width. However, it is also true that, if attention was the only factor influencing the representation of hands, a general improved representation should have also been found for finger lengths, which was not the case. Hence, it is unlikely that a general attentional mechanism only can explain these results.

To sum up, these results confirm that an embodied visual language can influence non-linguistic cognitive processes, indicating that mechanisms related to SL are not domain-specific and, instead, interact (Bettger et al. 1997). As seen with magicians (Cocchini et al. 2018), prolonged manual practice can produce long-term changes in the representation of the body. However, these changes may not be related to actual general improvement of the representation but appear modulated by the type of expertise (Coelho et al. 2019), space where the body action takes place, and the specific components of the body representation being measured (Brusa et al. 2021). Hence, the direction of distortions differs between expert groups, body parts, and body representation tasks.
